# Virulence Structure of *Blumeria graminis* f. sp. *tritici* and Its Genetic Diversity by ISSR and SRAP Profiling Analyses

**DOI:** 10.1371/journal.pone.0130881

**Published:** 2015-06-22

**Authors:** Na Liu, Z. Lewis Liu, Guoshu Gong, Min Zhang, Xu Wang, You Zhou, Xiaobo Qi, Huabao Chen, Jizhi Yang, Peigao Luo, Chunping Yang

**Affiliations:** 1 College of Agronomy, Sichuan Agricultural University, Chengdu, Sichuan, China; 2 National Center for Agricultural Utilization Research, Agricultural Research Service, U.S. Department of Agriculture, Peoria, Illinois, United States of America; 3 College of Resources, Sichuan Agricultural University, Chengdu, Sichuan, China; College of Agricultural Sciences, UNITED STATES

## Abstract

*Blumeria graminis* f. sp. *tritici*, which causes wheat powdery mildew, is an obligate biotrophic pathogen that can easily genetically adapt to its host plant. Understanding the virulence structure of and genetic variations in this pathogen is essential for disease control and for breeding resistance to wheat powdery mildew. This study investigated 17 pathogenic populations in Sichuan, China and classified 109 isolates into two distinct groups based on pathogenicity analysis: high virulence (HV, 92 isolates) and low virulence (LV, 17 isolates). Populations from Yibin (Southern region), Xichang (Western region), and Meishan (Middle region) showed lower virulence frequencies than populations from other regions. Many of the previously known resistance genes did not confer resistance in this study. The resistance gene *Pm*21 displayed an immune response to pathogenic challenge with all populations in Sichuan, and *Pm*13, *Pm*5b, *Pm*2+6, and *Pm*XBD maintained resistance. AMOVA revealed significantly higher levels of variation within populations and lower levels of variation among populations within regions. High levels of gene flow were detected among populations in the four regions. Closely related populations within each region were distinguished by cluster analyses using ISSR and SRAP alleles. Both ISSR and SRAP allele profiling analyses revealed high levels of genetic diversity among pathogenic populations in Sichuan. Although ISSR and SRAP profiling analysis showed similar resolutions, the SRAP alleles appeared to be more informative. We did not detect any significant association between these alleles and the virulence or pathogenicity of the pathogen. Our results suggest that ISSR and SRAP alleles are more efficient for the characterization of small or closely related populations versus distantly related populations.

## Introduction

Wheat powdery mildew is a destructive disease worldwide that is caused by *Blumeria graminis* (DC.) Speer f. sp. *tritici* emend. É. J. Marchal. In favorable climates, this pathogenic fungus infects the entire leaf surface, causing leaves to wither and resulting in severe yield losses. In Sichuan Province, an important wheat-producing region in southwest China, the disease generally causes yield losses of 5 to 8%. In severe epidemics, local farms have reported yield losses of up to 100% [1]. Growing disease-resistant cultivars is a common practice for disease management. However, new versions of pathogens often emerge rapidly and overcome host resistance [2]. Because of the geographically distinct climates in Sichuan, pathogens can over-summer in cool climates with altitudes above 595 m. The pathogen populations that over-summer on volunteer wheat plants can serve as an infection source and can attack wheat plants at lower attitudes, potentially causing gene flow and reconstruction of the pathogen population [1].

To achieve an efficient wheat powdery mildew disease resistance breeding program, it is necessary to understand the virulence frequency of powdery mildew and identify a source of resistance genes (*Pm* genes). Recently, due to high selective pressure from improper application and arrangement of resistant cultivars, the population structure of this pathogen has undergone rapid changes, and frequent shifts in the avirulence genes have occurred. This has led to loss-of-function of mutations in the corresponding *Pm* genes in China, including *Pm1* and the *Pm3* series [3–5]. Meanwhile, the virulence structure of the *B*. *graminis* f. sp. *tritici* population has also been affected by geographic distribution [5]. Although wheat powdery mildew populations have been widely studied, associations between the genetic backgrounds of wheat powdery mildew populations and their virulence in southwest China have not been explored. This lack of knowledge hinders disease control and genetic engineering efforts toward the development of disease-resistant cultivars.

The frequency of virulence to prevalent resistance genes is typically determined by the conventional evaluation method using a set of wheat cultivars; however, the ability of this method to detect genetic backgrounds that mediate the virulence of the pathogen is limited [6]. To understand the genetic diversity of wheat powdery mildew, reliable molecular evidence is needed. Historically, numerous DNA-based tools have been applied in population studies of plant pathogens, such as random amplified polymorphic DNA (RAPD), simple sequence repeats (SSRs), restriction fragment length polymorphisms (RFLPs) and amplified fragment length polymorphisms (AFLPs) [7–11]. However, most of these tools have some limitations in population genetics [12]. The recently developed inter-simple sequence repeats (ISSRs) and sequence-related amplified polymorphisms (SRAPs) methods are becoming popular approaches for the characterization of populations of numerous organisms, including fungi [13–18]. ISSRs have been demonstrated to be useful for analyzing the genetic diversity of a wide range of fungal species, including *Lentinula edodes*, *Agaricus*, *Fusarium poae*, and *Blumeria graminis* f. sp. *tritici* [19–22]. The simple, reproducible and high-throughput SRAP method targets amplification of open reading frames (ORFs) [23–25]. It has been widely used in research on genetic diversity, genetic linkage mapping and comparative genomic analyses in plants, including *Erianthus arundinaceum*, *Dactylis glomerata* L., *Piper* spp., *Lycium ruthenicum* Murr., *Hedychium* spp., *Pisum sativum* L., *Brassica* and *Arabidopsis* [26–31]. SRAP markers have also been used to analyze the genetic diversity of fungi, such as *Coprinus comatus*, *Polyporus umbellatus*, *Sclerotinia sclerotiorum*, *Puccinia striiformis* f. sp. *tritici* and *Tricholoma matsutake* [25, 32–35]. In a recent study involving genetic mapping of wheat powdery mildew, SRAP markers were commonly presented in many *Pm*-gene carrying cultivars [36, 37]. However, at the molecular level, the genetic diversity of powdery mildew populations is not well known.

We investigated the virulence of *B*. *graminis* f. sp. *tritici* from Sichuan, southwest China, against 30 isogenic wheat lines. We also characterized the genetic diversity of *B*. *graminis* f. sp. *tritici* using SRAP and ISSR methods. Here, we report the most updated virulence structure of wheat powdery mildew and present our discoveries regarding gene flow caused by epidemics of the pathogen in southwest China. This study also reveals candidate genes that may be useful in efficient disease resistance breeding programs.

## Materials and Methods

### Sample collection

Wheat leaves showing symptoms of powdery mildew were collected from 40 counties and subsequently grouped based on 16 administrative cities (Fig A in S1 File, Table B in S1 File). Because of the distinct geography in the Renshou region, which is administratively governed by Meishan City, samples collected from Renshou region were treated as an independent population. In total, we examined 17 population groups distributed throughout the Western, Southern, Middle, and Northeastern regions of Sichuan Province (Fig A in S1 File). The entire sampling area is located between approximate latitudes of 26°03’ to 34°19’ and longitudes of 97°21’ to 108°31’. The sampling site in southwest China was variable with respect to topography and included plains, basins, and mountainous regions. To avoid bias in sample collection, diseased leaves were taken from susceptible wheat cultivars in a random pattern, as previously reported [38]. To avoid contamination, each diseased leaf sample was placed in a clean paper bag. Samples were transferred to the laboratory for immediate isolation and purification of the pathogen. The Department of Plant Pathology at Sichuan Agricultural University is an advisory agent for plant disease management. Therefore, no specific permissions were required for the plant disease survey, including the collection of diseased samples from these defined regions. This study does not involve any endangered or protected species. All supplemental files have been combined into a single file: S1 File

### Isolation, purification, and maintenance of the pathogen

Healthy wheat seedlings were prepared for the isolation, purification, and maintenance of pathogenic isolates. Seeds of a susceptible wheat cultivar (Chuanyu 20) were placed in 75% alcohol for 3 min, washed 3 times with sterile water, and then placed on filter papers in a petri dish. The seed-containing petri dish was incubated in a growth chamber for 1–2 days until germination. Germinated seeds were transferred into soil-containing bottles (2.5 cm × 20 cm) with 5–7 seeds/bottle. The seeded bottle was sealed with Parafilm to avoid contamination and incubated in a growth chamber with controlled cycles of 14 h of continuous light at 18°C followed by 10 h of darkness at 16°C.

A purified isolate was obtained from each sample using the previously reported single spore purification method [39, 40]. Briefly, a piece of a freshly detached Chuanyu 20 leaf segment was placed on a piece of filter paper in a petri dish containing 50 μg/ml benzimidazole. Using a swab, we transferred a single pustule from a diseased sample onto the surface of a detached piece of fresh leaf segment. The leaf segment was incubated in a growth chamber with the light and temperature conditions described above. This procedure was repeated every 10 days until a single-colony isolate was obtained after three to four cycles. A purified culture was maintained, and fresh inoculum was produced on leaves from healthy Chuanyu 20 plants grown in bottles containing soil under the conditions described above.

### Virulence analysis

Virulence was examined in 30 isogenic lines harboring known disease resistance genes (Table 1). For each purified isolate, host plant responses were evaluated based on the gene-for-gene hypothesis [6, 40]. Seeds from the tested cultivars were maintained in a growth chamber under the conditions described above. Fresh leaf segments (3 cm) were obtained from the central regions of 10-day-old seedling leaves. Two susceptible cultivars, Chancellor and Funo, were used for control measurements as previously described [41, 42]. All differential isogenic lines and susceptible controls were inoculated with conidia isolated from a single spore and placed on a piece of filter paper in a petri dish containing 50 μg/ml benzimidazole. After inoculation, the petri dishes were maintained in a growth chamber as described above. A set of differentials and susceptible control were inoculated in a single petri dish with each isolate, and a minimum of three replications were performed. The leaf segments were scored 12 days after inoculation using a previously reported system [43] with modifications (Table A in S1 File). Using our more stringent evaluation standard, isolates on plant leaves showing immune responses (rated as 0) and resistance responses (rated as I) were considered to be avirulent, whereas isolates showing increased levels of mycelium and conidiospore production (rated as II, III and IV) were considered to be virulent. In addition, the frequency of occurrence of the corresponding genes for virulence in *B*. *graminis f*. *sp*. *tritici* was also calculated. The avirulent and virulent types observed were transformed into a binary coding matrix for computational analysis. Dendrograms were constructed by performing an unweighted pair-group analysis with arithmetic averages (UPGMA) using NTSYS-pc version 2.10 [44].

**Table 1:** Frequency of virulence of *Blumeria graminis* f. sp. *tritici* in Sichuan, China.

No.	Cultivar	Isogene	Number of virulent isolates	Frequency of virulence (%)
1	Funo	*¡ª*	109	100
2	Chancellor	*¡ª*	109	100
3	XX186	*Pm19*	99	90
4	Asosan /8cc	*Pm3a*	96	88
5	Coker 747	*Pm6*	96	88
6	Timgalen	*Pm6*	95	87
7	Axminster/8cc	*Pm1*	94	86
8	Chul /8cc	*Pm3b*	93	85
9	Hope /8cc	*Pm5*	88	80
10	Kolibri	*Pm3d*	83	76
11	CI14189	*Pm7*	80	73
12	Armada	*Pm4b*	80	73
13	W150	*Pm3e*	78	71
14	Khapli /8a	*Pm4a*	76	69
15	Mish Amber/8cc	*Pm3f*	75	68
16	Normandie	*Pm1+2+9*	68	62
17	Baimian 3	*Pm4+8*	66	60
18	Sonora/8cc	*Pm3c*	65	59
19	Amigo	*Pm17*	61	55
20	Mission	*Pm4b+5b*	59	54
21	Kavkaz	*Pm8*	54	49
22	Maris Dove	*Pm2+MLD*	42	38
23	Ulka /8cc	*Pm2*	36	33
24	Coker 983	*Pm5+6*	36	33
25	Era	*PmEra*	31	28
26	Xiaobaidong	*PmXBD*	21	19
27	Maris Huntsman	*Pm2+6*	20	18
28	Aquila	*Pm5b*	18	16
29	R4A	*Pm13*	13	11
30	Yangmai5/Sub.6v	*Pm21*	0	0

### DNA isolation and PCR

Genomic DNA was extracted with a fungal DNA kit (Omega Bio-Tek, Norcross, GA, USA) following the manufacturer’s protocol. A total of 20 primers that produced clearly distinguishable and reproducible fragments were selected and used in this study for ISSR and SRAP assays (Table 2). For the ISSR assay, we prepared a PCR containing 10 μl 2× Power Tap PCR Master Mix, 8 μl ddH_2_O, 1 μl primer and 1 μl template DNA. The amplification protocol for the ISSR assay was as follows: 94°C for 5 min (pre-denaturation), followed by 40 cycles of 94°C for 30 sec, 45–58°C for 45 sec and 72°C for 2 min, with a final extension at 72°C for 7 min. The amplification products were separated on a 1.5% agarose gel.

**Table 2:** Primers used for SRAP and ISSR analysis in this study.

Assay	ID	Sequence (5’-3’)
SRAP	Me1F	TGAGTCCAAACCGGATA
	Me2F	TGAGTCCAAACCGGAGC
	Me3F	TGAGTCCAAACCGGAAT
	Me4F	TGAGTCCAAACCGGACC
	Me5F	TGAGTCCAAACCGGAAG
	Me6F	TGAGTCCAAACCGGTAG
	Me7F	TGAGTCCAAACCGGTTG
	Me8F	TGAGTCCAAACCGGGTGT
	Em1R	GACTGCGTACGAATTAAT
	Em2R	GACTGCGTACGAATTTGC
	Em3R	GACTGCGTACGAATTGAC
	Em4R	GACTGCGTACGAATTTGA
	Em5R	GACTGCGTACGAATTAAC
	Em6R	GACTGCGTACGAATTGCA
	Em7R	GACTGCGTACGAATTATG
	Em8R	GACTGCGTACGAATTAGC
ISSR	808	AGAGAGAGAGAGAGAGC
	807	AGAGAGAGAGAGAGAGT
	812	GAAGAGAGAGAGAGAGA
	841	GAAGAGAGAGAGAGAAGYC
	891	HVHTGTGTGTGTGTGTG
	887	DVDTCTCTCTCTCTCTC
	890	VHVGTGTGTGTGTGTGT
	888	BDBCACACACACACACA
	884	HBHAGAGAGAGAGAGAG
	835	AGAGAGAGAGAGAGAGYC

B = C, G, or T; D = A, G, or T; H = A, C, or T; V = A, C, or G.

For the SRAP assay, the PCR mix consisted of 10 μl 2× Power Tap PCR Master Mix, 7 μl ddH_2_O, 1 μl each of the F and R primers, and 1 μl template. The SRAP amplification protocol was as follows: 94°C for 5 min (pre-denaturation); 5 cycles of 94°C for 1 min, 35°C for 1 min, and 72°C for 1.5 min; 35 cycles of 94°C for 1 min, 50°C for 1 min, and 72°C for 1.5 min; and a final extension of 72°C for 7 min. The amplification products were separated on a 2% agarose gel.

### Data analysis

ISSR and SRAP data for each DNA sample were coded as 1 (presence) or 0 (absence) to produce a binary matrix, and dendrograms were constructed by UPGMA using NTSYS-pc version 2.10 [44]. Genetic diversity analysis was performed using PopGene 32 software [45]. The parameters calculated for genetic diversity included the total number of bands (*TNB*), the number of polymorphic bands (*NPB*), the percentage of polymorphic bands (*PPB*), the observed number of alleles (*Na*), the effective number of alleles (*N*e), Shannon’s information index (*I*), Nei’s (1973) gene diversity (*H*), and gene flow (*N*m). The polymorphic information content (PIC) and the resolving power (Rp) of the primers were evaluated using previously described formulas [46, 47]. Components of genetic variance within and among populations were estimated by analysis of molecular variance (AMOVA) using Arlequin3.0 [48]. Matrix data derived from the ISSR and SRAP methods were compared using the Mantel test with GenAlEx 6.501 at 999 permutations (Mantel, 1967) [49–51].

## Results

### Virulence

#### Virulence frequency

A total of 109 purified pathogenic isolates of *B*. *graminis* f. sp. *tritici* were obtained from 327 samples of infected leaves collected from 40 counties representing 17 populations in Sichuan, China (Fig A in S1 File, Table B in S1 File). Of the 28 previously identified resistance genes, *Pm21* (harbored by Yangmai5/Sub.6v) displayed a unique immune response to all 109 isolates tested (Table 1). It was the only gene able to maintain a resistance response during challenge with all pathogenic isolates in Sichuan. The virulence frequency was less than 20% in four isogenes: *Pm13*, *Pm5b*, *Pm2+6*, and *PmXBD* (harbored by cultivars R4A, Aquila, Maris Huntsman, and Xiaobaidong) (Table 1). The virulence frequencies were greater than 60% for previously recognized resistance genes, including *Pm1*, *Pm2*, *Pm3a*, *Pm3b*, *Pm3d*, *Pm3e*, *Pm3f*, *Pm4a*, *Pm4b*, *Pm5*, *Pm6*, *Pm7*, *Pm9*, and *Pm19* (Table 1). The remaining genes displayed intermediate levels of resistance or susceptibility with virulence frequencies between 20% and 60%.

**Fig 1:**
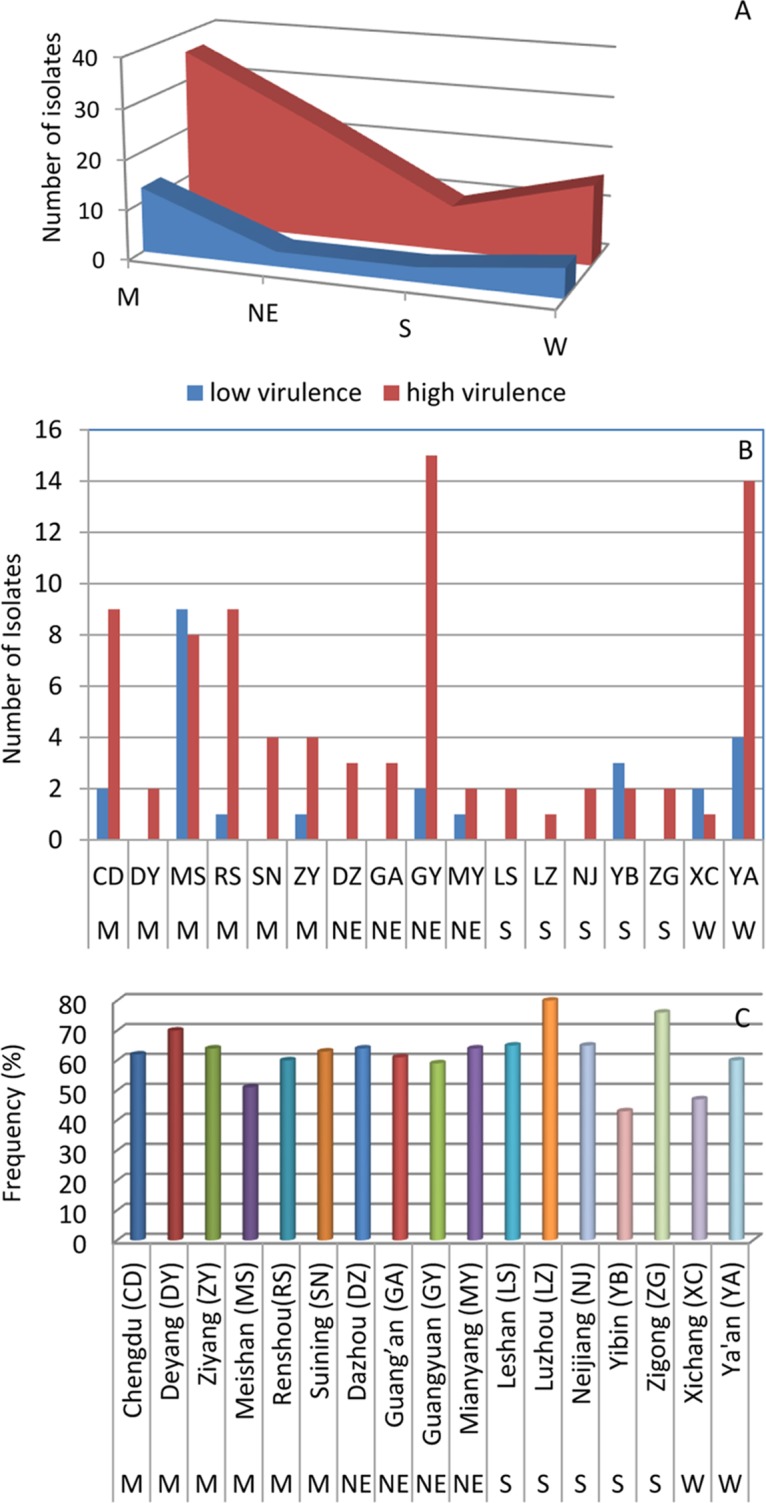
Virulence distribution of *Blumeria graminis* f. sp. *tritici* in Sichuan, China.

Many isolates from the Middle region displayed high levels of virulence (Fig 1A); however, some isolates from this region displayed low virulence. Most of the resistance genes appeared to be distributed in the Meishan population (MS) (Fig 1B). The number of genes for virulence were varied significantly among regional populations, ranging from 4 to 26 genes (Table 3). The Southern region had the highest number of genes, with a frequency of up to 80% in Zigong (ZG) and Luzhou (LZ) (Table 3, Fig 1C). In this high-virulence region, the frequency of virulence in the Yibin population (YB) was 43%, which was significantly lower than that in other populations in the region. Overall, for regional comparison, the Western region showed the lowest distribution of virulence, as represented by Xichang (XC) and Ya’an (YA). Another notable population with a lower gene frequency was identified in Meishan (MS) in the Middle region. Although populations in the Middle region showed high virulence frequencies, the number of resistance genes was also relatively higher, especially in the Meishan region, compared with that observed in other regions.

**Table 3:** The occurrence frequency of the corresponding genes for virulence in *Blumeria graminis* f. sp. *tritici* in Sichuan, China.

Regions	Number of genes ^a^	Frequency (%)
Luzhou (LZ)	24	80
Zigong (ZG)	22~24	76
Deyang (DY)	20~22	70
Leshan (LS)	16~23	65
Neijiang (NJ)	17~22	65
Mianyang (MY)	14~24	64
Dazhou (DZ)	17~22	64
Ziyang (ZY)	11~24	64
Suining (SN)	15~24	63
Chengdu (CD)	13~26	62
Guang’an (GA)	16~20	61
Ya'an (YA)	11~22	60
Renshou (RS)	4~22	60
Guangyuan (GY)	8~23	59
Meishan (MS)	6~24	51
Xichang (XC)	11~20	47
Yibin (YB)	6~19	43

^a^ Number of detected genes for virulence of *Blumeria graminis* f. sp. *tritici*.

#### Cluster analysis of virulence

**Fig 2:**
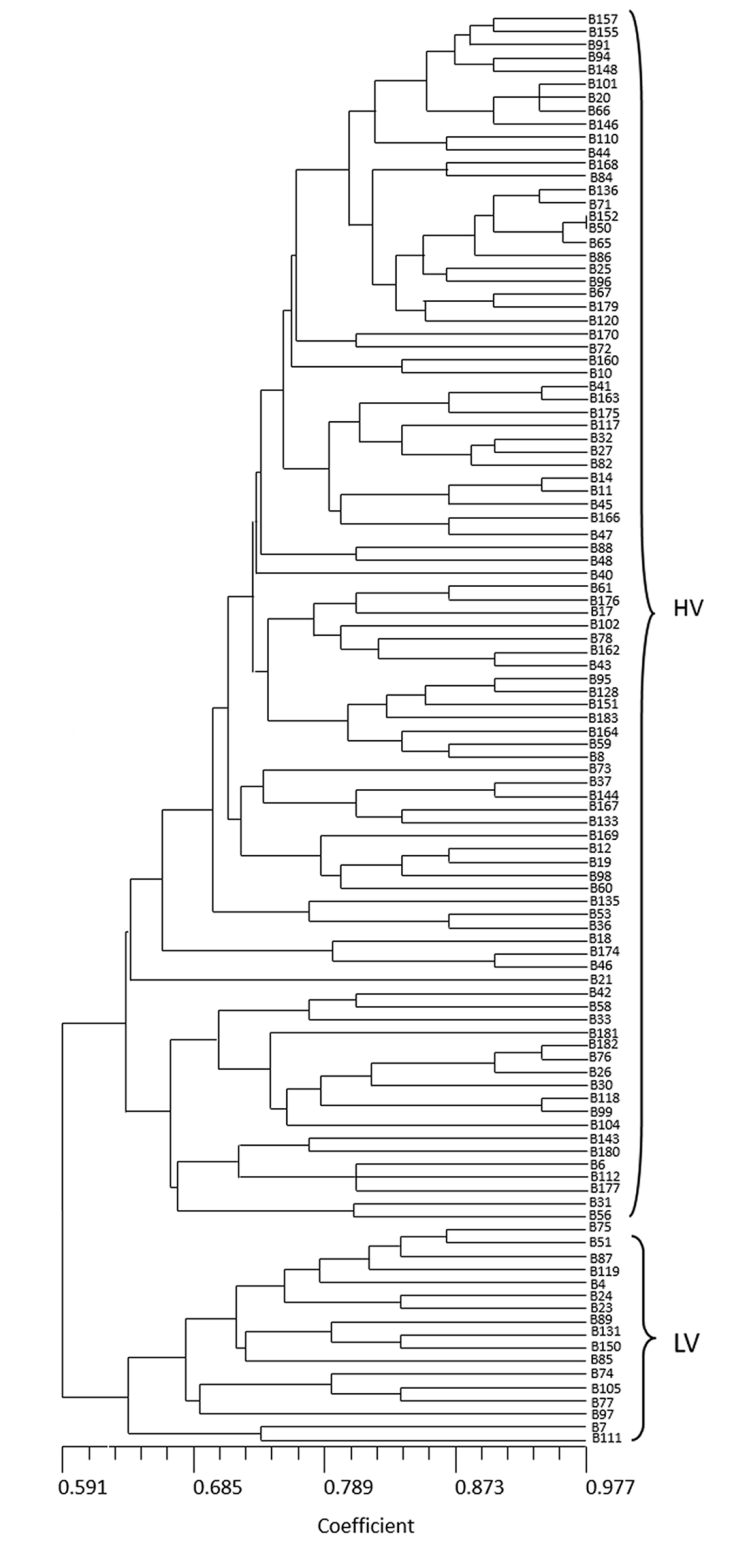
UPGMA dendrogram of *Blumeria graminis* f. sp. *tritici* strains based on the virulence data.

Based on pathogenicity, cluster analysis was performed to sort the 109 purified isolates from throughout Sichuan province into two distinct groups. At a similarity coefficient cutoff of 0.63, 92 isolates fell into a large cluster with a high frequency of virulence (63.19%). This group was designated the high-virulence (HV) group (Fig 2). The low-virulence (LV) group consisted of 17 isolates and showed an average virulence frequency of 38.63%. The LV group included nine isolates from the Middle region, four isolates from the Western region, three isolates from the Northeastern region, and one isolate from the Southern region (Fig 2, Table C in S1 File). In this group, there was a close relationship between isolates B4, 51, 75, 87, and 119, which displayed a significantly lower frequency of virulence than other isolates in the LV group. The lowest virulence frequency was 0.13% for isolate B51 from Renshou in the Middle region. The Middle region also appeared to have more sources of resistance. Regionally, isolates from the Meishan population showed relatively lower virulence frequencies compared with isolates from other populations (Fig 1, Table C in S1 File).

### Genetic diversity

#### ISSR amplification

**Fig 3:**
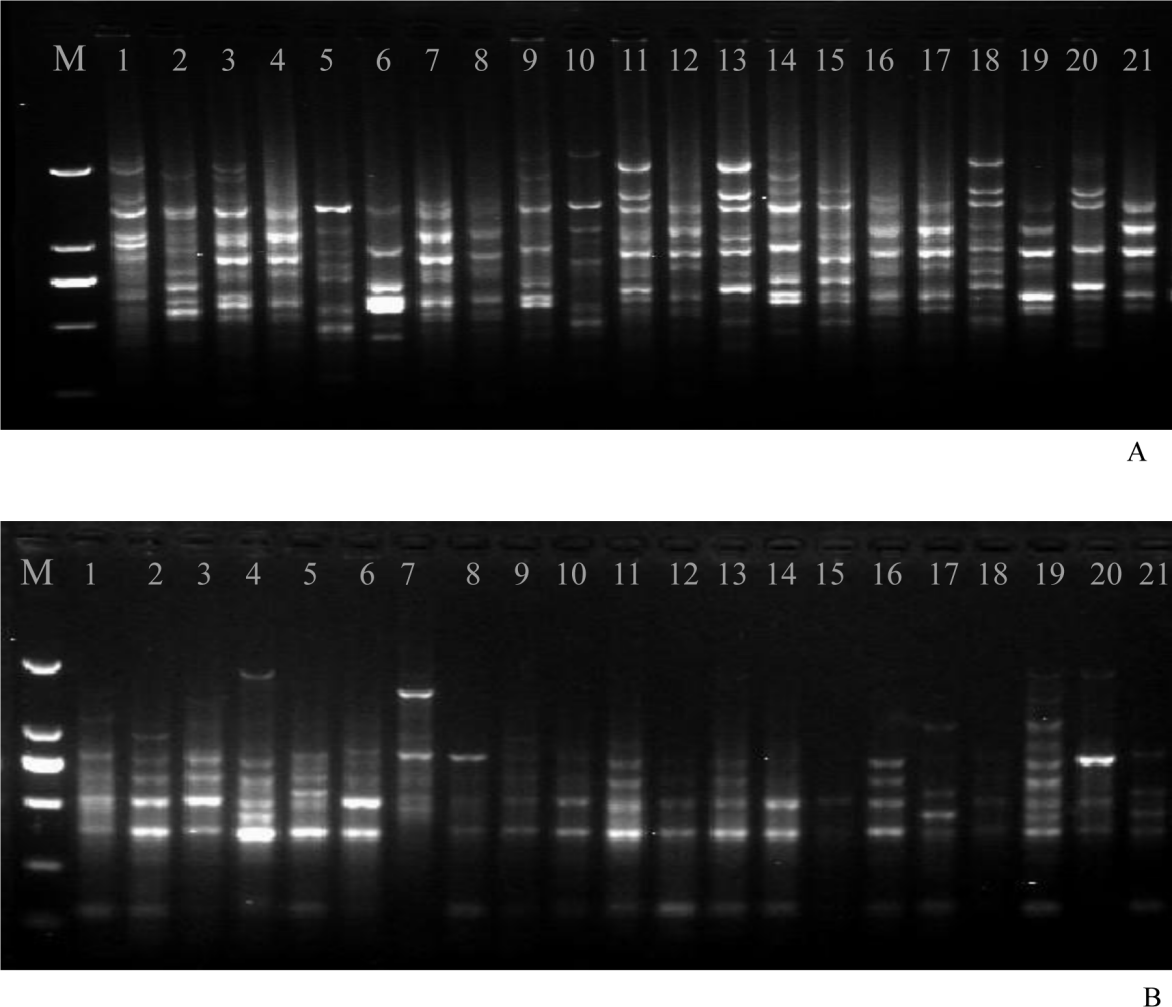
Representative molecular marker profile. (A): ISSR profiles in the tested 21 strains of *Blumeria graminis* f. sp. *tritici* with primer 807. (B): SRAP profiles in the tested 21 strains of *Blumeria graminis* f. sp. *tritici* with primer Me2/Em8.

DNA from each of 105 isolates was amplified using the ISSR method. A total of 186 clearly distinguishable amplification fragments ranging from 100 to 2,000 bp were obtained using 10 ISSR primers (Fig 3A). Among these, 135 fragments were polymorphic, with an average of more than 13 polymorphic bands for each primer. ISSR primers 835 and 808 were the most efficient, producing 16 and 15 polymorphic bands, respectively. The lowest number of bands was obtained from primer 890, which generated 11 polymorphic bands. DNA fragments derived from this primer also displayed the highest PIC score of 0.41 (Table 4). The rates of informative polymorphic bands from ISSR amplifications varied from 65% to 80%, with PIC scores ranging from 0.32 to 0.41.

**Table 4:** PCR amplification of *Blumeria graminis* f. sp. *tritici* isolates using ISSR and SRAP primers.

Assay	Primer	Amplified bands	Polymorphic bands	Percentage of polymorphic bands (%)	PIC	RP	Average RP
ISSR	808	19	15	78.95	0.36	10.37	0.55
	807	19	14	73.68	0.35	10.71	0.56
	812	20	13	65.00	0.33	10.35	0.52
	841	16	11	68.75	0.32	7.39	0.46
	891	15	11	73.33	0.35	7.98	0.53
	887	21	15	71.43	0.33	10.25	0.49
	890	14	11	78.57	0.41	9.54	0.68
	888	21	15	71.43	0.34	10.19	0.49
	884	21	14	66.67	0.33	10.52	0.50
	835	20	16	80.00	0.35	10.31	0.52
	Average	18.6	13.5	72.78	0.35	9.76	0.53
SRAP	Me6/Em8	16	13	81.25	0.33	7.31	0.46
	Me8/Em8	16	11	68.75	0.33	8.04	0.50
	Me5/Em5	12	5	41.67	0.24	3.62	0.30
	Me1/Em1	16	13	81.25	0.35	8.50	0.53
	Me8/Em6	12	9	75.00	0.32	5.54	0.46
	Me2/Em8	15	10	66.67	0.35	7.83	0.52
	Me8/Em4	15	12	80.00	0.36	8.14	0.54
	Me4/Em8	19	13	68.42	0.29	7.77	0.41
	Me5/Em8	19	12	63.16	0.29	7.44	0.39
	Me3/Em8	14	9	64.29	0.29	5.75	0.41
	Average	15.4	10.7	64.29	0.32	6.99	0.45

PIC, polymorphic information content; RP, resolving power.

#### SRAP amplification

A total of 154 bands ranging from 100 to 2,000 bp were obtained from 10 SRAP primers (Fig 3B). Among these, 107 bands were polymorphic, with an average of 10.7 bands per primer. Primers Me6/Em8 and Me4/Em8 generated the highest number of polymorphic bands, and primer set Me5/Em5 produced the lowest number of informative bands. The polymorphism scores for primer sets Me5/Em5, Me6/Em8 and Me1/Em1 were 41.7%, 81.3% and 81.3%, respectively (Table 4). These primers had an average PIC of 0.314, ranging from 0.236 to 0.349. The primer pairs Me2/Zm8, Me6/Zm8, Me8Zm8, Me8Zm4, Me1/Zm1, and Me8/Zm6 all had PIC values greater than 0.30, while the primer pairs Me6/Em8, Me1/Em1, and Me8/Em4 generated the most polymorphic information and had the highest PIC scores, ranging from 0.33 to 0.36.

#### Cluster analysis

**Fig 4:**
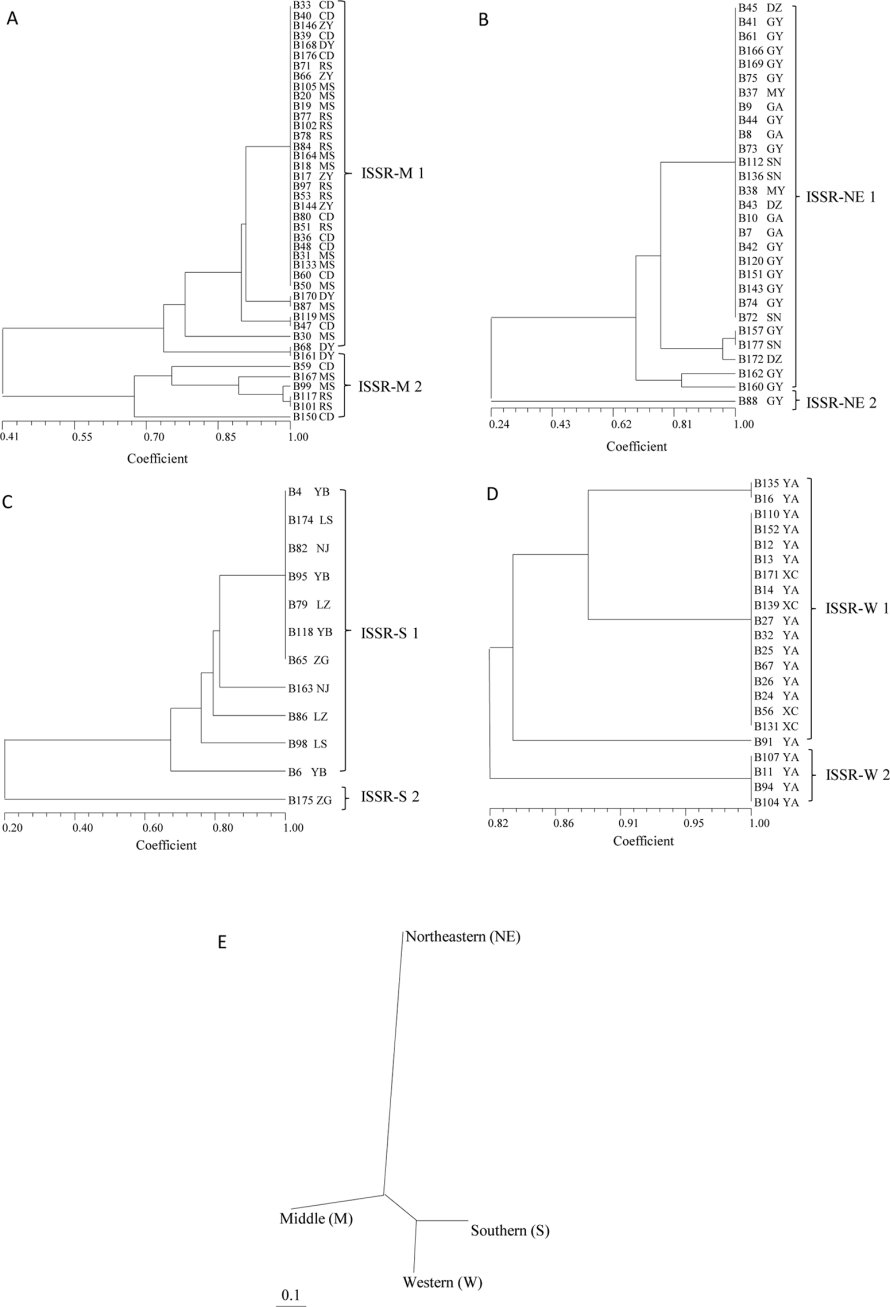
UPGMA dendrogram of *Blumeria graminis* f. sp. *tritici* strains based on the ISSR data showing relationships among strains isolated from the Middle region (A), Northeastern region (B), Southern region (C), Western region (D); Regional relationships (E) are also shown.

Cluster analysis based on Nei’s unbiased genetic distances was performed using ISSR profiling for 105 isolates collected from all regions in Sichuan. Three groups (ISSR1, ISSR2 and ISSR3) were established from the 105 isolates. ISSR1 contained 94 isolates that can be divided into two subgroups: ISSR1-1 and ISSR1-2 (Fig B in S1 File). However, groupings based on ISSR data reflected neither virulence/pathogenicity nor the geographical origin of the isolates. We conducted additional analyses based on the regional origin of the isolates and obtained four more informative trees for each region (Fig 4A–4D). From the 42 isolates from the Middle region, two distinct groups were formed: the ISSR-M1 group contained 36 isolates, and the ISSR-M2 group contained 6 isolates (Fig 4A). The 6 isolates in the ISSR-M2 group were from Meishan, Renshou, and Chengdu, whereas the ISSR-M1 group included isolates from all locations in the Middle region. Of the 29 isolates from the Northeastern region, one unique isolate from Guanyuan, B88, was clearly separated and represented a single isolate group, ISSR-NE2. The other 28 isolates from this region essentially clustered into one group: ISSR-NE1 (Fig 4B). Similarly, in the Southern region, 12 isolates were clustered into two groups (ISSR-S1 and ISSR-S2), whereas the B175 isolate from Zigong formed a unique group (ISSR-S2) (Fig 4C). Isolates from the Western region were more similar, forming only two subgroups (ISSR-W1 and ISSR-W2) (Fig 4D). Thus, isolates from the Western region displayed lower diversity than isolates from all other regions. The regional variations in ISSR alleles in isolates from the Northeastern region were distinct from those of isolates from all other regions (Fig 4E).

**Fig 5:**
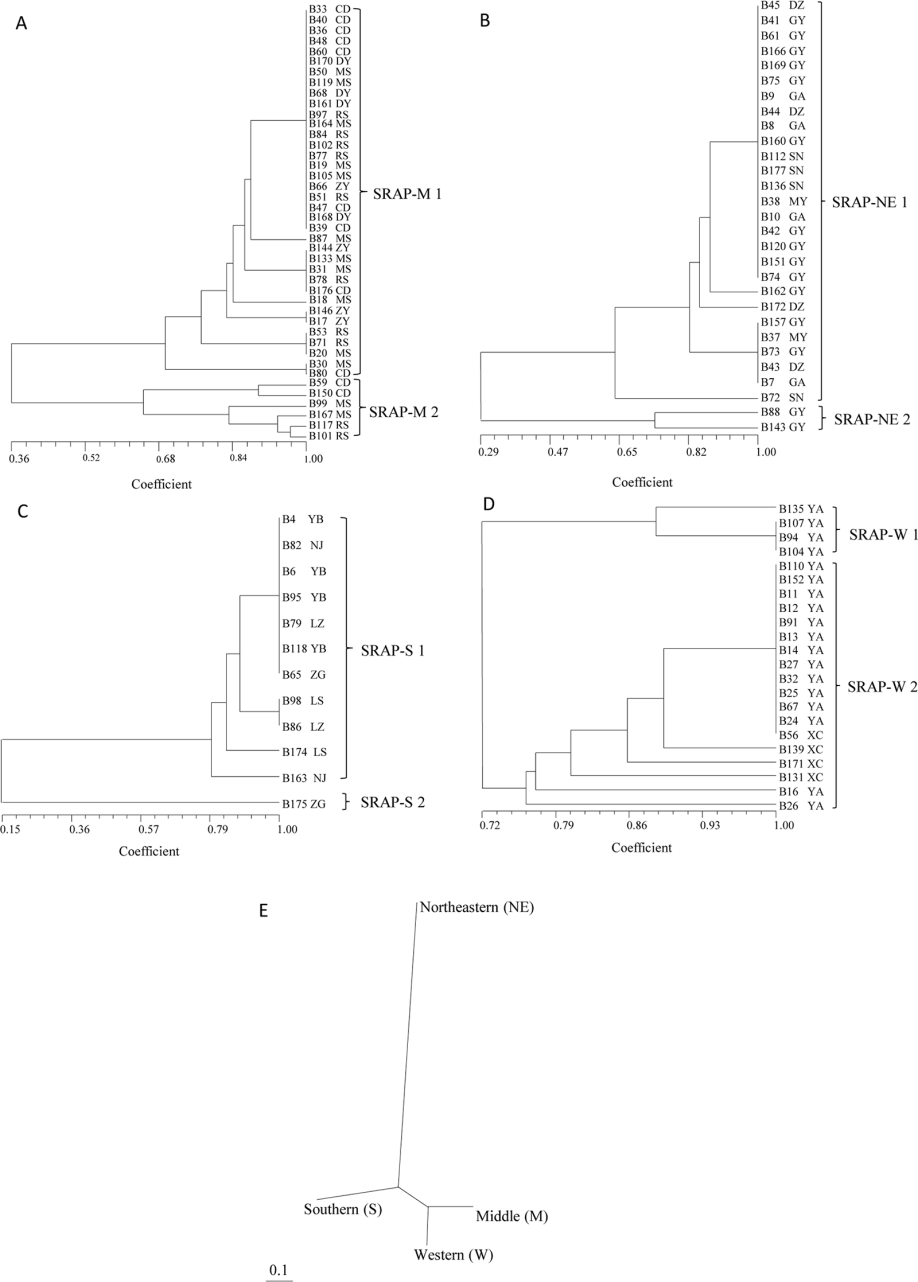
UPGMA dendrogram of *Blumeria graminis* f. sp. *tritici* strains based on the SRAP data showing relationships among strains isolated from the Middle region (A), Northeastern region (B), Southern region (C), Western region (D); Regional relationships (E) are also shown.

For the data obtained from SRAP profiling, we performed the same series of analyses used on the ISSR data. The UPGMA dendrogram derived from SRAP profiling of 105 isolates revealed three groups: SRAP1, SRAP2 and SRAP3. The SRAP1 group was further separated into four subgroups (Fig C in S1 File). There were no overlaps or close relationships between the SRAP and ISSR groupings. There were also no close relationships to virulence or to the geographical origin of the isolates. Regional analysis identified two subgroups in the Middle region (SRAP-M1 and SRAP-M2), two subgroups in the Northeastern region (SRAP-NE-1 and SRAP-NE2), two subgroups in the Southern region (SRAP-S1 and SRAP-S2), and two subgroups in the Western region (SRAP-W1 and SRAP-W2) (Fig 5A–5D). Unlike the different outcomes derived from SRAP and ISSR analyses of the entire group of isolates, groupings of isolates at the regional level showed similar results with both methods. In general, clusters from each region contained the same member of isolates. However, variations in alleles between SRAP and ISSR were clearly observed among the isolates. For example, isolates B86 and B98 in group SRAP-S1 from the Southern region were identical in the SRAP analysis but different in the ISSR analysis (Figs 4C and 5C). In other regions, similar patterns were observed in the analysis of isolates within the same group. There was a high level of variation in the SRAP and ISSR alleles for all regions except the Western region. However, the pathogenicity or virulence of isolates within groupings did not appear to be closely related to their group assignments. In the regional comparisons, the ISSR and SRAP analyses of allele polymorphisms revealed similar relationships among regional populations with little variation. Both analyses indicate that Northeastern populations are distantly related to populations from the other regions (Figs 4E and 5E).

We also pooled the ISSR and SRAP data of all 105 isolates and performed a combined analysis. The resulting UPGMA dendrogram had low resolution power (Fig D in S1 File). Isolate classifications in the combined analysis showed no close relationship to either the ISSR or SRAP analysis. There was no correlation between the ISSR and SRAP matrices, as indicated by the low correlation coefficient (r = 0.438) generated by the Mantel test (Mantel 1967).

#### Genetic diversity analysis

There were high levels of genetic diversity in the 17 populations in Sichuan based on both ISSR and SRAP analyses. On a regional basis, the ISSR method suggested that there were higher levels of variation in the Middle region compared with the other regions (Table 5). On the other hand, the SRAP method suggested that there was more variation for populations in the Western region; however, the Middle region had the highest observed number of alleles (*Na*) based on SRAP analysis. Both ISSR and SRAP analysis showed that populations in the Southern region had the least variation as measured by the observed number of alleles (*Na*), the effective number of alleles (*Ne*), Nei’s genetic diversity (*H*), and Shannon’s information index (*I*) (Table 5).

**Table 5:** Parameters related to the genetic diversity and gene flow of *Blumeria graminis* f. sp. *tritici* in Sichuan, China.

Regions	ISSR					SRAP				
	*Na*	*Ne*	*H*	*I*	*Nm*	*Na*	*Ne*	*H*	*I*	*Nm*
Middle (M)	1.984	1.621	0.352	0.520	2.148	1.955	1.480	0.291	0.445	1.851
West (W)	1.930	1.558	0.325	0.485	2.880	1.948	1.508	0.306	0.465	3.991
Northeast (NE)	1.909	1.532	0.313	0.470	1.526	1.890	1.496	0.295	0.446	1.686
South (S)	1.823	1.525	0.306	0.454	0.448	1.812	1.455	0.274	0.415	0.401
Average	1.911	1.559	0.324	0.482	—	1.901	1.485	0.291	0.443	—
Among regions	—	—	—	—	7.984	—	—	—	—	8.126

*Na*, observed number of alleles; *Ne*, effective number of alleles [Kimura and Crow (1964)]; *H*, Nei’s (1973) gene diversity; *I*, Shannon’s information index [Lewontin (1972)]; *Nm*, gene flow. Mean values of *Na*, *Ne*, *H*, *I* are presented in this table.

The gene flow among the four regions was estimated to be 7.984 by ISSR and 8.126 by SRAP analysis (Table 5). Our data suggest that there were high levels of gene migration among these regions. Considering gene flow within each region, the Southern region showed the lowest level of gene flow, estimated at 0.448 and 0.401 using ISSR and SRAP alleles, respectively. Compared with the Southern region, the Northeastern region had a higher rate of gene flow, estimated at 1.526 and 1.686 using ISSR and SRAP alleles, respectively. The rate of gene flow for Middle populations was estimated at 2.148 by ISSR and at 1.851 by SRAP. The Western region had the highest level of gene flow at 2.88 and 3.991, as determined by ISSR and SRAP, respectively. These results suggest that pathogenic populations migrated within the Western region. The results of AMOVA showed significantly high levels of variation within the 17 populations. In contrast, variation among the four regions was less significant. The variation among populations within a region was slightly higher than the variation among regions (Table 6).

**Table 6:** Analysis of molecular variance (AMOVA) based on ISSR and SRAP data.

	Source of variation	df	Sum of squares	Variance components	Percentage variation	P value
ISSR	Among regions	3	157.383	0.233	0.72%	<0.0001
	Among populations within regions	13	514.523	1.742	5.34%	<0.0001
	Within populations	88	2694.037	30.614	93.94%	<0.0001
	Total	104	3365.943	32.589		
SRAP	Among regions	3	121.472	0.431	1.74%	<0.0001
	Among populations within regions	13	352.876	0.695	2.82%	<0.0001
	Within populations	88	2073.766	23.566	95.44%	<0.0001
	Total	104	2548.114	24.691		

## Discussion

Using 109 purified pathogenic isolates of *B*. *graminis* f. sp. *tritici* representing 17 populations in Sichuan, China, we identified two distinct virulence groups: HV and LV. In addition, we revealed an updated virulence structure for populations in the Middle, Northeastern, Southern, and Western regions of Sichuan. By applying ISSR and SRAP molecular markers, we also characterized the genetic diversity of the pathogen and detected high levels of genetic variation among populations in Sichuan. Our results suggest that ISSR and SRAP alleles can be used independently to study closely related populations, although these alleles do not reveal significant associations with the virulence or pathogenicity of the pathogen.

Traditionally, a visible mycelium or conidiospore has been considered to be a symptom of an avirulent response. In this study, we used a more stringent standard for disease evaluation, and we rated such symptoms as a virulent response. We believe that the evaluation standards used in this study provide a more realistic view of the host response that will support the development of more efficient disease resistance breeding programs. Using this standard, we found that the resistance gene *Pm*21 had an immune response against all pathogenic isolates. Low virulence frequencies were detected for the genes *Pm*13, *Pm*5b, *Pm*2+6, and *Pm*XBD. However, many previously identified resistance genes lose their function against pathogens with higher virulence frequencies, including *Pm*1, *Pm*2, *Pm*3a, *Pm*3b, *Pm*3d, *Pm*3e, *Pm*3f, *Pm*4a, *Pm*4b, *Pm*5, *Pm*6, *Pm*7, *Pm*9 and *Pm*19. Such pathogens accounted for more than 80% of the pathogenic isolates investigated. These results highlight the potential for epidemics under favorable conditions [1, 52].

*B*. *graminis* f. sp. *tritici* is a highly variable wheat pathogen, and its virulence frequencies are influenced by resistance genes expressed in cultivars grown in the field [40, 53, 54]. High virulence frequencies were observed for *Pm*5, *Pm*3a, *Pm*3b and *Pm*3d, indicating the high prevalence of these genes in the host cultivars examined. Conversely, resistance genes with low virulence frequencies, such as *Pm*13, *Pm*5b, and *Pm*2+6, were either rarely present or completely absent among the cultivars investigated. Consistent planting of a single cultivar over time leads to the loss of resistance in that cultivar, which results in rapid and frequent breakdown of disease resistance, making disease management challenging [55].

High virulence frequencies were observed for many genes in this study, confirming that these populations had successfully adapted to the host resistance genes. Isolates in the Middle region had high levels of virulence, and populations in the Southern region contained the highest numbers of genes for virulence, with frequencies of up to 80%. Due to the loss of disease resistance, the corresponding isogenic cultivars should no longer be used for large-scale planting or disease resistance breeding programs in these regions. On the other hand, *Pm*13, *Pm*5b, *Pm*2+6, and *Pm*XBD retained a higher level of resistance to powdery mildew as indicated by lower virulence frequencies. Thus, these resistance genes are strong candidates for wheat breeding programs. *Pm*21, a commonly introduced resistance gene in China [56], triggered a unique immune response to all pathogenic isolates in Sichuan. We also demonstrated that virulence responses vary by geographic location. For example, populations in the Southern region generally had high virulence frequencies, whereas populations in Yibin displayed the lowest virulence frequencies in this region. Regardless of geographic differences, we believe that the resistance gene *Pm*21 remains the top candidate for use in disease resistance breeding programs. Because high levels of virulence were observed in the Middle and Southern populations, we recommend the introduction of a new source of resistance in these regions. In addition, a close monitoring of any dynamic changes in the virulence structure is necessary.

Polymorphism analyses derived from ISSR and SRAP profiles revealed that 105 isolates of *B*. *graminis* f. sp. *tritici* from 17 populations formed three groups. A reasonable level of polymorphic information was observed, ranging from 65% to 80% for ISSRs and 42% to 81% for SRAPs. These levels are similar to the levels found in previous reports [57–61]. No close relationships were detected by ISSR or SRAP analyses of the entire population. Because ISSR profiling detects random inter-simple sequence repeats in the genome and SRAP profiling reflects sequence variation within gene coding regions, there is no overlap between the principles of the two methods. Attempts to combine the two methods to identify taxonomic entities have produced inconclusive results [60, 62]. Similarly, our attempt to combine the two methods did not improve the resolution. The results of this study suggest that ISSR and SRAP methods can be used independently, but not in combination, to characterize closely related populations as needed.

In contrast to the inconsistent grouping of the total isolates by ISSR and SRAP alleles, cluster analysis by either technique generates similar isolate classifications for populations that are closely related at the regional level. The isolates belonging to groups derived from ISSR and SRAP alleles were identical in the Middle and Southern regions. Groupings of isolates in the Northeastern and Western regions were also very similar. However, the relationships among isolates within the group were different in terms of allele variations between SRAPs and ISSRs with different principles. Groupings by these methods did not appear to have any relationship with the virulence of the isolates, which is consistent with previous observations [54, 63]. It is unlikely that these molecular markers are involved in gene functions related to virulence or pathogenicity. Notably, close relationships were identified among isolates in the SRAP-M2 and ISSR-M2 groups from Chengdu, Meishan, and Renshou using both methods (Figs 4A and 5A). Although there were allele variations, the close relationship between isolates from the same geographic location was clear using both methods. This suggests that ISSR and SRAP methods may be more efficient for characterizing small or closely related populations than for distantly related populations. AMOVA revealed significantly higher levels of variation within populations than among populations within regions, indicating a higher level of genetic diversity but a lower level of genetic divergence in Sichuan. These data also suggest that high levels of genetic overlap are the result of extensive gene flow. Gene flow and differentiation are important factors in evaluating the genetic structure of a population [64]. The estimated gene flow among the four regions was moderate, with *Nm* = 7.984 and 8.126 by ISSR and SRAP analysis, respectively. This suggests that gene migration and long-distance pathogen transference occurred among these four regions. A high level of gene flow was observed in the Western region between Xichang (altitude > 1500 m) and Ya’an (altitude ~ 595–775 m), representing long-distance gene migration over more than 240 km. Wheat powdery mildew was reportedly transferred from high to low altitude areas by wind [65], and long-distance disease transference has been previously observed [66–68]. In this study, a greater number of genes for virulence were detected in the Southern region compared to the other regions. However, the rate of gene flow within the Southern region was low, indicating persistent local evolution within this region.

In Sichuan, wheat is a major crop and is planted in large areas. The uniquely diverse geography and climate in southwestern China yield conditions that are favorable for wheat powdery mildew growth, transference, and the resultant epidemics. Our study highlights the potential threat of this disease in major wheat-producing regions of China. Understanding the genetic diversity of the pathogen is essential for the development of efficient disease control programs. The novel candidate resistance genes and the data regarding the virulence structure and population diversity of this pathogen that were presented in this study will support more focused efforts in the management of wheat powdery mildew.

## Supporting Information

S1 File Standards for classifying the virulence of wheat powdery mildew in wheat seedling stages (**Table A**). Characteristics of the isolates used in this study (**Table B**). The frequency of genes for virulence of 17 powdery mildew isolates in the low-virulence group (**Table C**). Locations at which wheat leaves infected with powdery mildew were collected (**Fig A**). A UPGMA dendrogram of *Blumeria graminis* f. sp. *tritici* strains based on ISSR data (**Fig B**). A UPGMA dendrogram of *Blumeria graminis* f. sp. *tritici* strains based on SRAP data (**Fig C**). A UPGMA dendrogram of *Blumeria graminis* f. sp. *tritici* strains based on combined data from ISSR and SRAP markers (**Fig D**).
(PDF)
