# Supplementary material for: Virulence Structure of Blumeria graminis f. sp. tritici and Its Genetic Diversity by ISSR and SRAP Profiling Analyses
Source: PLoS One. 2015 Jun 22;10(6):e0130881. doi: 10.1371/journal.pone.0130881 (PMC4476801; doi:10.1371/journal.pone.0130881)
Supplement: S1 File — Standards for classifying the virulence of wheat powdery mildew in wheat seedling stages (Table A). Characteristics of the isolates used in this study (Table B). The frequency of genes for virulence of 17 powdery mildew isolates in the low-virulence group (Table C). Locations at which wheat leaves infected with powdery mildew were collected (Fig A). A UPGMA dendrogram of Blumeria graminis f. sp. tritici strains based on ISSR data (Fig B). A UPGMA dendrogram of Blumeria graminis f. sp. tritici strains based on SRAP data (Fig C). A UPGMA dendrogram of Blumeria graminis f. sp. tritici strains based on combined data from ISSR and SRAP markers (Fig D). (PDF) [file pone.0130881.s001.pdf]

Table A: Standards for classifying the virulence of wheat powdery mildew in wheat seedling stages

Evaluation	Infection types	Symptom descriptions
Avirulent	0	Immune (I): no visible sign of infection or necrosis, no mycelium.
	1	Resistant (R): increasing from no mycelium to little mycelium, sometimes with necrosis.
Virulent	2	Moderately resistant (MR): increasing amount of mycelium, little conidiospore production with necrosis.
	3	Moderately compatible (MS): large amount of mycelium, moderate conidiospore production.
	4	Completely compatible (HS): large amount of mycelium, substantial conidiospore production.

Table B: Characteristics of the isolates used in this study

No.	Code	Source	Latitude	Longitude	C-1	C-2	No.	Code	Source	Latitude	Longitude	C-1	C-2
1	B41	Donghe, Wangcang, Guangyuan	32°14'55"	106°18'08"	I1-2	S1-1	62	B167	Liusheng, Dongpo, Meishan	29°55'02"	103°55'44"	I1-1	S1-2
2	B61	Hanyang, Jiange, Guangyuan	32°08'03"	105°30'51"	I1-2	S1-1	63	B87	Liusheng, Dongpo, Meishan	29°55'03"	103°55'46"	I1-1	S1-4
3	B73	Donghe, Wangcang, Guangyuan	32°13'46"	106°19’03"	I1-2	S1-2	64	B105	Yongshou, Dongpo, Meishan	29°57'44"	103°50'19"	I1-1	S1-4
4	B75	Donghe, Wangcang, Guangyuan	32°13'36"	106°17'22"	I1-2	S1-1	65	B20	Yongshou, Dongpo, Meishan	29°57'43"	103°50'22"	I1-1	S2
5	B88	Hanyang, Jiange, Guangyuan	32°08'24"	105°30'31"	I1-2	S1-2	66	B19	Yongshou, Dongpo, Meishan	29°57'47"	103°50'15"	I2	S1-4
6	B157	Donghe, Wangcang, Guangyuan	32°13'43"	106°17'20"	I1-2	S1-1	67	B31	Liusheng, Dongpo, Meishan	29°55'04"	103°55'44"	I2	S1-2
7	B166	Shengli, Yuanba ,Guangyuan	32°20'30"	105°57'41"	I1-2	S1-1	68	B164	Yongshou, Dongpo, Meishan	29°57'43"	103°50'12"	I3	S1-3
8	B169	Donghe, Wangcang, Guangyuan	32°13'26"	106°17'14"	I1-2	S1-1	69	B76	Hongwuxing, Fuxing, Meishan	29°56'07"	103°52'13"	na	na
9	B162	Shengli, Yuanba, Guangyuan	32°20'31"	105°57'42"	I1-2	S1-1	70	B89	Hongwuxing, Fuxing, Meishan	29°56'11"	103°52'16"	na	na
10	B160	Shengli, Yuanba, Guangyuan	32°20'30"	105°57'41"	I1-2	S1-2	71	B128	Liusheng, Dongpo, Meishan	29°55'04"	103°55'43"	na	na
11	B42	Donghe, Wangcang, Guangyuan	32°13'52"	106°17'20"	I1-2	S2	72	B183	Hongwuxing, Fuxing, Meishan	29°56'05"	103°52'11"	na	na
12	B74	Donghe, Wangcang, Guangyuan	32°13'37"	106°17'16"	I1-2	S2	73	B53	Fujia, Renshou, Meishan	29°59'45"	104°08'01"	I1-1	S1-4
13	B120	Donghe, Wangcang, Guangyuan	32°13'54"	106°17'19"	I1-2	S2	74	B71	Manjing, Renshou, Meishan	29°55'27"	104°08'56"	I1-1	S1-4
14	B151	Donghe, Wangcang, Guangyuan	32°13'36"	106°17'22"	I1-2	S2	75	B51	Fujia, Renshou, Meishan	29°59'42"	104°08'07"	I1-2	S1-4
15	B143	Donghe, Wangcang, Guangyuan	32°13'43"	106°17'10"	I1-2	S2	76	B84	Fujia, Renshou, Meishan	29°59'45"	104°08'02"	I1-2	S1-3
16	B180	Donghe, Wangcang, Guangyuan	32°13'64"	106°17'07"	na	na	77	B78	Fujia, Renshou, Meishan	29°59'43"	104°08'11"	I1-2	S1-3
17	B181	Hanyang, Jiange, Guangyuan	32°08'15"	105°30'57"	na	na	78	B102	Fujia, Renshou, Meishan	29°59'46"	104°08'08"	I1-2	S1-3
18	B37	Songya, Youxian, Mianyang	31°38'99"	104°82'74"	I1-2	S1-1	79	B97	Manjing, Renshou, Meishan	29°55'22"	104°08'57"	I1-2	S1-2
19	B38	Songya, Youxian, Mianyang	31°38'98"	104°82'67"	I1-2	S1-4	80	B117	Manjing, Renshou, Meishan	29°55'41"	104°08'60"	I1-2	S1-2
20	B155	Songjiang, Youxian, Mianyang	31°38'96"	104°82'85"	na	na	81	B101	Manjing, Renshou, Meishan	29°55'42"	104°08'32"	I1-2	S1-2
21	B182	Songjiang, Youxian, Mianyang	31°38'00"	104°82'84"	na	na	82	B77	Fujia, Renshou, Meishan	29°59'43"	104°08'08"	I2	S1-3
22	B44	Dongba,Tongchuan, Dazhou	31°18'52"	107°31'61"	I1-2	S1-2	83	B11	Baishi, Tianquan, Ya’an	30°04'51"	102°46'26"	I1-1	S1-2
23	B45	Zhuxian, Pujia, Dazhou	31°34'22"	107°52'62"	I1-2	S1-1	84	B12	Baishi, Tianquan, Ya’an	30°04'53"	102°46'22"	I1-1	S1-2
24	B172	Guancheng, Tongzhou, Dazhou	31°12'51"	107°30'15"	I1-2	S1-1	85	B13	Baishi, Tianquan, Ya’an	30°04'52"	102°46'27"	I1-1	S1-2
25	B43	Dongba, Tongchuan, Dazhou	31°18'57"	107°31'56"	I1-2	S2	86	B27	Dongcheng, Yucheng, Ya’an	29°98’04"	102°97’97"	I1-1	S1-4
26	B68	Taipingsi, Luojiang, Deyang	31°18'57"	104°33'37"	I1-2	S1-2	87	B32	Dongcheng, Yucheng, Ya’an	29°98’07"	102°97’88"	I1-1	S1-4
27	B161	Taipingsi, Luojiang, Deyang	31°18'58"	104°33'44"	I1-2	S1-2	88	B24	Dongcheng, Yucheng, Ya’an	29°98’16"	102°97’99"	I1-1	S1-4
28	B170	Longfeng, Jingyang, Deyang	31°09'27"	104°27'00"	I1-2	S1-2	89	B25	Dongcheng, Yucheng, Ya’an	29°98’07"	102°97’87"	I1-1	S1-4
29	B168	Huaguang, Luojiang, Deyang	31°19'06"	104°34'26"	I1-2	S2	90	B67	Chenghoulu, Yucheng, Ya’an	29°98’66"	102°99’24"	I1-2	S1-4
30	B177	Shehong, Suining	30°52'17"	105°38'93"	I1-2	S1-2	91	B16	Chenghoulu, Yucheng, Ya’an	29°98’63"	102°99’26"	I1-2	S1-2
31	B72	Shehong, Suining	30°86'84"	105°38'62"	I1-2	S1-1	92	B94	Chenghoulu, Yucheng, Ya’an	29°98’57"	102°99’21"	I1-2	S1-2
32	B112	Chuanshan, Suining	30°47'91"	105°60'71"	I1-1	S1-2	93	B26	Chenghoulu, Yucheng, Ya’an	29°98’61"	102°99’20"	I1-2	S1-4
33	B136	Chuanshan, Suining	30°47'93"	105°60'49"	I1-1	S1-4	94	B107	Qingyuan, Yucheng, Ya’an	29°98’66"	102°98’79"	I1-2	S1-1
34	B10	Zhenhe, Zaoshan, Guang’an	30°29'28"	106°41'20"	I1-2	S2	95	B135	Qingyuan, Yucheng, Ya’an	29°98’59"	102°98’78"	I1-2	S1-1
35	B7	Zhaizi, Hu’an, Guang’an	30°29'28"	106°41'20"	I1-2	S2	96	B110	Qingyuan, Yucheng, Ya’an	29°98’67"	102°98’75"	I1-2	S1-1
36	B8	Liuqiao, Daishi, Guang’an	30°31'04"	106°48'44"	I1-2	S1-2	97	B152	Qingyuan, Yucheng, Ya’an	29°98’63"	102°98’73"	I1-2	S1-1
37	B9	Liuqiao, Daishi, Guang’an	30°31'07"	106°48'40"	I3	S1-1	98	B91	Chenghoulu, Yucheng, Ya’an	29°98’59"	102°99’20"	I1-2	S1-2
38	B33	Guihu, Xindu, Chengdu	30°49'34"	104°09'35"	I1-2	S1-1	99	B14	Dongcheng, Yucheng, Ya’an	29°98’00"	102°97’96"	I1-2	S1-3
39	B36	Chongyang, Chongzhou,Chengdu	30°38'40"	103°41'07"	I1-2	S1-2	100	B104	Dongcheng, Yucheng,Ya’an	29°98’05"	102°97’95"	I1-2	S3
40	B40	Huanyuan, Xinjin, Chengdu	30°28'17"	103°53'28"	I1-2	S1-1	101	B21	Chenghoulu, Yucheng,Ya’an	29°98’58"	102°99’22"	na	na
41	B80	Hong’an, Longquan,Chengdu	30°41'29"	104°16'52"	I1-2	S1-2	102	B23	Chenghoulu, Yucheng,Ya’an	29°98’63"	102°99’24"	na	na
42	B48	Qingjiang, Jintang, Chengdu	30°54'46"	104°23'13"	I1-2	S1-2	103	B111	Qingyuan, Yucheng,Ya’an	29°98’64"	102°98’77"	na	na
43	B59	Qunlie, Shuangliu, Chengdu	30°37'51"	104°00'15"	I1-2	S1-2	104	B179	Qingyuan, Yucheng,Ya’an	29°98’52"	102°98’79"	na	na
44	B150	Qunlie, Shuangliu, Chengdu	30°37'47"	104°00'23"	I1-2	S1-3	105	B174	Dingzi, Jiajiang,Leshan	29°45'33"	103°30'56"	I1-2	S1-2
45	B60	Zhengxing, Shuangliu, Chengdu	30°26'33"	104°02'50"	I1-2	S1-2	106	B98	Dingzi, Jiajiang, Leshan	29°45'44"	103°30'35"	I1-2	S1-2
46	B39	Wenjing, Xinjin, Chengdu	30°29'25"	103°45'05"	I1-2	S2	107	B163	Tuojiang, Zizhong, Neijiang	29°27'24"	105°00'56"	I1-2	S1-2

47	B176	Wenjiang, Xinjin, Chengdu	30°29'19"	103°45'01"	I1-2	S2	108	B82	Huanyuantan, Shizhong, Neijiang	29°37'00"	105°01'38"	I1-2	S1-2
48	B47	Qingjiang, Jintang, Chengdu	30°54'45"	104°23'15"	I2	S2	109	B65	Yiqiao, Gaodong, Zigong	30°38'08"	104°75'87"	I3	S2
49	B46	Qingjiang, Jintang, Chengdu	30°54'44"	104°23'19"	na	na	110	B175	Jinming, Da’an, Zigong	29°21'47"	104°46'23"	I1-2	S1-2
50	B148	Zhengxing, Shuangliu, Chengdu	30°26'23"	104°02'44"	na	na	111	B79	Shuangsha, Guling, Luzhou	27°49'59"	105°45'17"	I1-2	S1-3
51	B85	Wenmingsi, Yanjiang, Ziyang	30°05'04"	104°40'37"	na	na	112	B86	Dazhai, Guling, Luzhou	27°49'59"	105°45'17"	I1-2	S1-4
52	B146	Huachao, Jianyang, Ziyang	30°16'12"	104°30'53"	I1-2	S1-1	113	B95	Haibin, Shaping, Yibin	28°47'30"	104°41'17"	I1-1	S1-3
53	B17	Huajia, Jianyang, Ziyang	30°16′26	104°26′30	I2	S1-1	114	B4	Bailian, Shaping, Yibin	28°47'28"	104°41'16"	I1-1	S1-1
54	B144	Huajia, Jianyang, Ziyang	30°16′22	104°26′35	I2	S1-1	115	B118	Bailian, Shaping, Yibin	28°47'26"	104°41'12"	I1-1	S1-4
55	B66	Wenmingsi, Yanjiang, Ziyang	30°05'09"	104°40'33"	I2	S1-4	116	B6	Haibin, Shaping, Yibin	28°47'28"	104°41'18"	I1-2	S1-2
56	B50	Wanguang, Wansheng, Meishan	30°05'03"	103°37'38"	I1-2	S1-2	117	B58	Siping, Pingshan, Yibin	28°67'51"	104°10'91"	na	na
57	B99	Wanguang, Wansheng, Meishan	30°05'05"	103°37'33"	I1-2	S1-2	118	B56	Mapingba, Xining, Xichang	27°95'18"	102°21'94"	I1-2	S1-3
58	B133	Wanguang, Wansheng, Meishan	30°05'04"	103°37'35"	I1-2	S1-2	119	B171	Mapingba, Xining, Xichang	27°95'30"	102°21'86"	I1-2	S1-2
59	B30	Liusheng, Dongpo, Meishan	29°55'05"	103°55'44"	I1-1	S1-2	120	B131	Mapingba, Xining, Xichang	27°95'34"	102°21'96"	I1-2	S1-3
60	B18	Yongshou, Dongpo, Meishan	29°57'42"	103°50'22"	I1-1	S1-3	121	B139	Gucheng, Qionghailu, Xichang	27°80'36"	102°31'88"	I3	S1-3
61	B119	Yongshou, Dongpo,, Meishan	29°57'39"	103°50'27"	I1-1	S1-2	122	B96	Gucheng, Qionghailu,Xichang	27°95'39"	102°31'97"	na	na

Note: C1: UPGMA Clustering by ISSR data, I=ISSR; C2: UPGMA Clustering by SRAP data, S=SRAP. "na" indicates not available.

Table C: The frequency of genes for virulence of 17 powdery mildew isolates in the low-virulence group

Region	Isolate	Population	Number of genes [a]	Gene frequency (%)	Average
Middle(M)	B150	CD	13	0.433	
	B87	MS	6	0.200	
	B119	MS	9	0.300	
	B105	MS	13	0.433	
	B89	MS	13	0.433	
	B51	RS	4	0.133	
	B77	RS	16	0.533	
	B97	RS	17	0.567	
	B85	ZY	11	0.367	0.378
Northeast(NE)	B7	GA	16	0.533	
	B75	GY	8	0.267	
	B74	GY	16	0.533	0.444
South(S)	B4	YB	6	0.200	0.200
West(W)	B131	XC	12	0.400	
	B24	YA	11	0.367	
	B111	YA	12	0.400	
	B23	YA	14	0.467	0.408

Note： [a] Number of detected genes for virulence of *Blumeria graminis* f. sp. *tritici*.

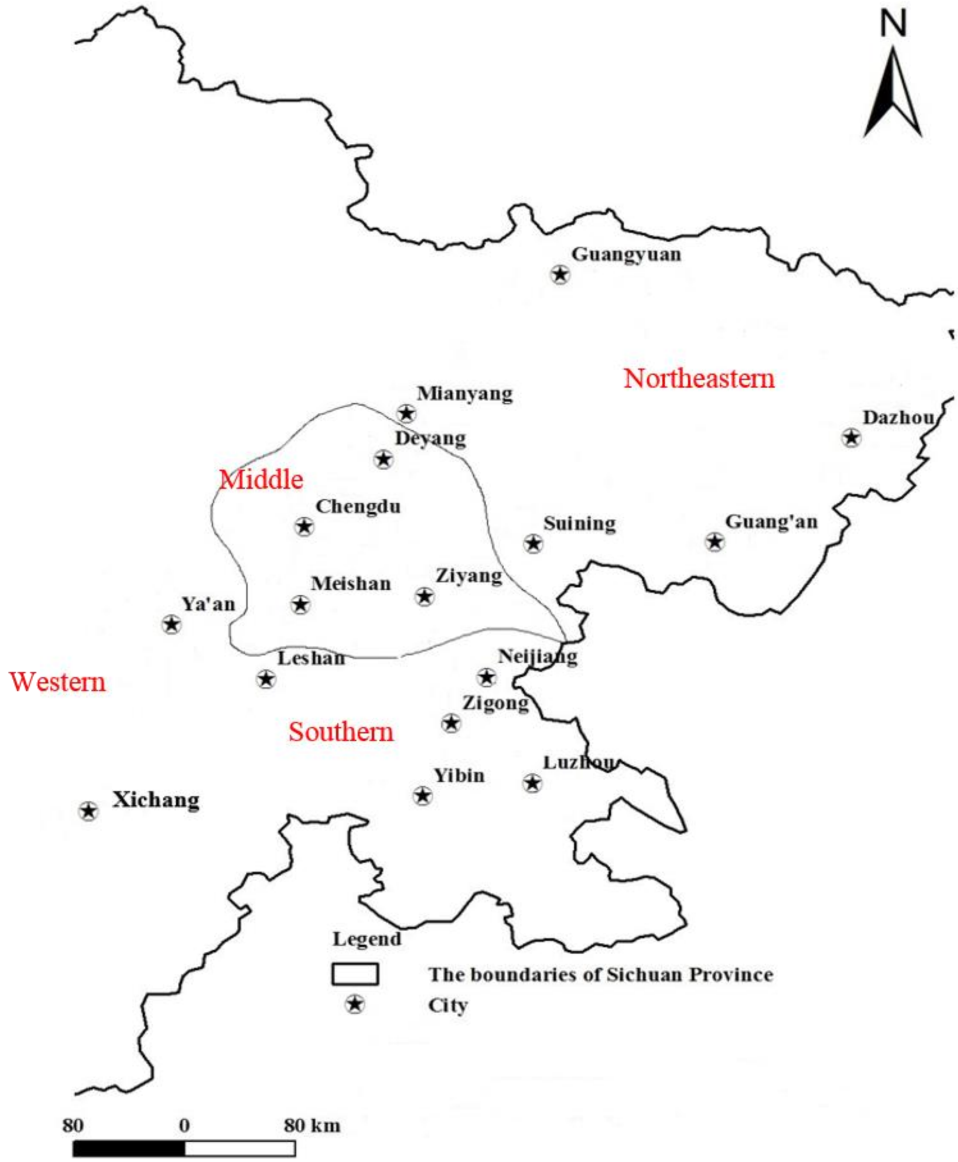

Fig. A: Locations at which wheat leaves infected with powdery mildew were collected.

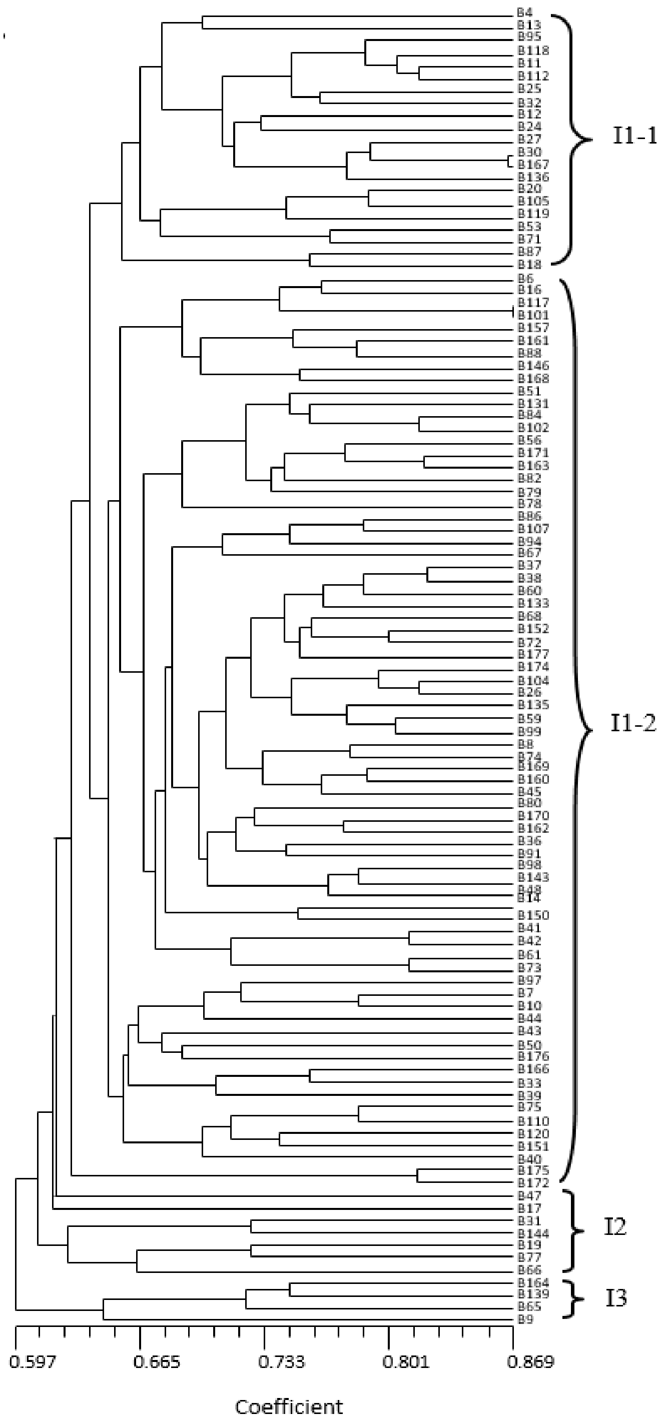

Fig. B: A UPGMA dendrogram of *Blumeria graminis* f. sp. *tritici* strains based on ISSR data.

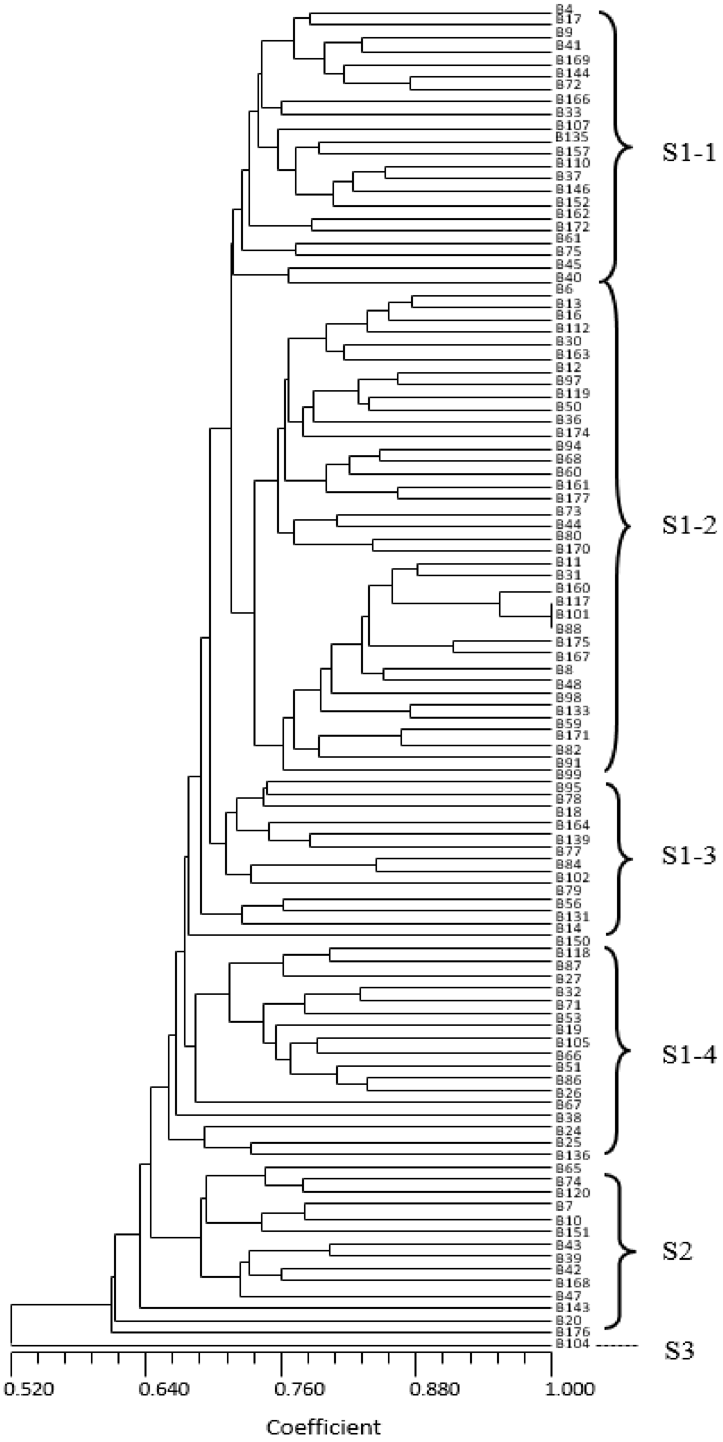

Fig. C: A UPGMA dendrogram of *Blumeria graminis* f. sp. *tritici* strains based on SRAP data.

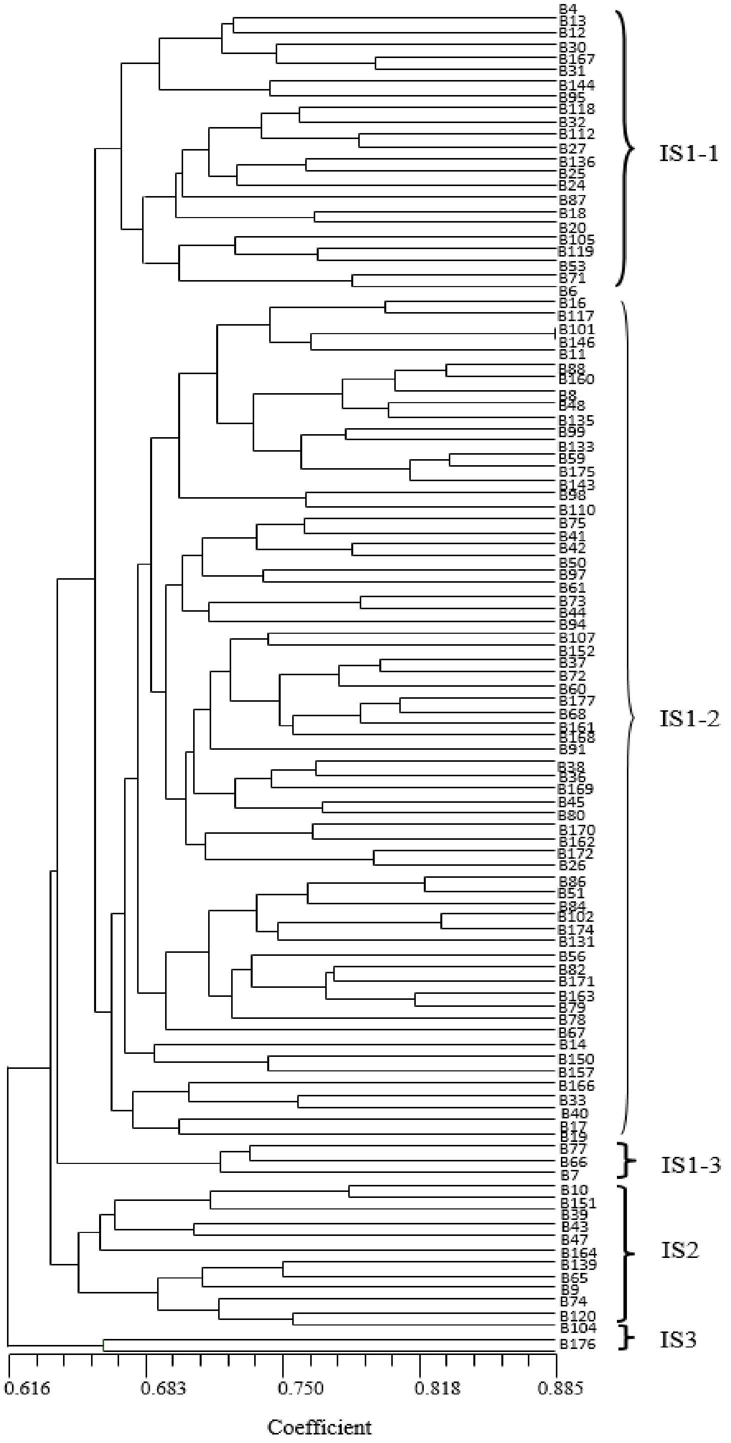

Fig. D: A UPGMA dendrogram of *Blumeria graminis* f. sp. *tritici* strains based on combined data from ISSR and SRAP markers.
